# Positional Role Differences in the Aerobic and Anaerobic Power of Elite Basketball Players

**DOI:** 10.1515/hukin-2015-0124

**Published:** 2015-12-30

**Authors:** Haris Pojskić, Vlatko Šeparović, Edin Užičanin, Melika Muratović, Samir Mačković

**Affiliations:** 1 Mid Sweden University, Faculty of Human Sciences, Department of Health Sciences, Sweden; 2 Center for Sports Excellence – Tuzla, Bosnia and Herzegovina; 3 Tuzla University, School of Physical Education and Sport, Bosnia and Herzegovina

**Keywords:** body composition, countermovement jump, fatigue index

## Abstract

The aim of the present study was to compare the aerobic and anaerobic power and capacity of elite male basketball players who played multiple positions. Fifty-five healthy players were divided into the following three different subsamples according to their positional role: guards (n = 22), forwards (n = 19) and centers (n = 14). The following three tests were applied to estimate their aerobic and anaerobic power and capacities: the countermovement jump (CMJ), a multistage shuttle run test and the Running-based Anaerobic Sprint Test (RAST). The obtained data were used to calculate the players’ aerobic and anaerobic power and capacities. To determine the possible differences between the subjects considering their different positions on the court, one-way analysis of variance (ANOVA) with the Bonferroni post-hoc test for multiple comparisons was used. The results showed that there was a significant difference between the different groups of players in eleven out of sixteen measured variables. Guards and forwards exhibited greater aerobic and relative values of anaerobic power, allowing shorter recovery times and the ability to repeat high intensity, basketball-specific activities. Centers presented greater values of absolute anaerobic power and capacities, permitting greater force production during discrete tasks. Coaches can use these data to create more individualized strength and conditioning programs for different positional roles.

## Introduction

Basketball is an intermittent, physically demanding sport with many defensive and offensive actions, requiring players to repeatedly engage in spells of intense activities (sprinting, shuffling, jumping, etc.) down the basketball court with some walking and slight jogging in between (Abdelkrim et al., 2007; McInnes et al., 1995). It is also a game with continuously changing pace, involving numerous fast and explosive applications of basketball techniques, such as rebounding, driving, lay-ups, jump shooting, shot blocking, fast breaks and high speed play, which incorporate aerobic and anaerobic energetic processes (Maud and Foster, 2006; Narazaki et al., 2009; Tessitore et al., 2006). It has been reported that elite basketball players spend 75% of their playing time with a heart rate greater than 85% of its maximum value (McInnes et al., 1995). Consequently, to play the sport at the highest level, players must have optimally developed levels of explosive power (Hoffman et al., 1996; Struzik et al., 2014; Štrumbelj et al., 2014), agility (Hoffman et al., 1996; Abdelkrim et al., 2010; Delextrat and Cohen, 2008), aerobic power (Abdelkrim et al., 2007, 2010; McInnes et al., 1995; Narazaki et al., 2009; Štrumbelj et al., 2014), anaerobic power (Hoffman et al., 1996; Delextrat and Cohen, 2008; Struzik et al., 2014) and anaerobic capacities (Apostolidis et al., 2004).

Depending on the position (guard, forward or center), players develop different physical fitness levels as well as different body compositions and morphological profiles that determine their role on the court. Guards are usually the shortest and fastest players on the team with the best ball handling ability, while centers are the tallest and slowest players on the team. Recent studies have shown that centers are taller and heavier with a higher body fat content than guards and forwards (Jeličić et al., 2002; Ostojić et al., 2006; Pojskić et al., 2014; Sallet et al., 2005). At the same time, guards have better aerobic and anaerobic capacities (Abdelkrim et al., 2010; Gocentas et al., 2011; Köklü et al., 2011; Latin et al., 1994), as well as speed and agility (Abdelkrim et al., 2010; Köklü et al., 2011), while forwards and centers are better in muscular strength and absolute power (Abdelkrim et al., 2010; Köklü et al., 2011; Ostojić et al., 2006).

Basketball is a sport discipline with a high anaerobic component dominant in intensive activities while the aerobic component prevails during active rest and activities of low intensity (Abdelkrim et al., 2007; McInnes et al., 1995). Blood lactate concentration in basketball has been reported to be between 3.7 and 13.2 mmol/l, with large variability among playing position (Abdelkrim et al., 2007; McInnes et al., 1995; Rodriguez–Alonso et al., 2003). Moreover, the involvement of aerobic power in basketball players is greater than was first thought (Abdelkrim et al., 2007; McInnes et al., 1995; Narazaki et al., 2009), indicating a need for aerobic and anaerobic testing of basketball players, as well as testing and comparing players at different positions.

Unfortunately, to the best of our knowledge, there is lack of studies performed on Bosnian elite basketball players. Therefore, we tested players from four Bosnian professional basketball teams. Two types of immediate (short-term) anaerobic energy systems were indirectly assessed. The adenosine triphosphate system tested the players’ ability to perform vertical, countermovement jumps (CMJ) without an arm swing, which had been found to be a very reliable tool for assessing athletes’ lower limb explosive power (Bosco et al., 1983; Markovic et al., 2004). Furthermore, the CMJ without an arm swing was reported to be a good predictor of the jumping abilities of basketball players when performing the jump shot (Struzik et al., 2014). Another test for the evaluation of the phosphocreatine system by the running based anaerobic sprint test (RAST) was found to be a valid (Zacharogiannis et al., 2004) and reliable tool (Zagatto et al., 2009), as well as a very practical test to assess the anaerobic index and excellent hyperlactatemia inductor (de Araujo et al., 2014). Basketball players perform approximately 200 high intensity movements during a game with intermittent and variable recovery periods, which can result in elevated lactate concentration (Abdelkrim et al., 2007). Therefore, the application of the RAST that induces hyperlactatemia as a means to test the utilization of the anaerobic metabolism has emerged as a valuable measure of these athletes. The aerobic system was evaluated with the multistage shuttle run test. The prediction of VO2max by this test was reported to be very reliable (Leger and Gadoury, 1989).

The purpose of the study was to examine differences in aerobic and anaerobic performance among guards, forwards and centers. We hypothesized differences in performance among these three subgroups. A secondary purpose was to examine aerobic and anaerobic performance of elite Bosnian basketball players.

## Material and Methods

### Participants

Fifty-five healthy basketball players (age: 19.09 ± 3.13 years; body height: 189.13 ± 8.30 cm; body mass: 83.42 ± 12.48 kg) from four teams of the Bosnian Premier League voluntarily participated in the study. The sample was divided into three groups according to the playing position. All of them were healthy without any history of neuromuscular diseases or reported injury in the previous six months. At the time of the study, they had 7.5 ± 2.6 years of competitive experience. According to their clubs’ head coaches, they trained 10 hours a week (5 sessions of 2 hours each) on the court, improving their technical and tactical skills, and 4.5 hours a week (3 sessions of 1.5 hours each) off the court, in the gym, improving their strength, power and endurance, with a basketball game played every Saturday or Sunday. They were asked to refrain from heavy training, tobacco, alcohol and caffeine use and to avoid sleep deprivation for at least two days prior to the testing sessions. The subjects were allowed to consume a light meal at least three hours prior the beginning of testing. To stay properly hydrated, the players were asked not to drink substantial volume at once; instead, they were required to drink water often, in small amounts, during testing. Players were told they were free to withdraw from the study at any time without consequences. Written informed consent was received from all players after a detailed verbal and written explanation of the experimental design, the purpose of the study, testing protocols, research benefits and potential risks of the study was provided. The study was approved by the Tuzla University Ethics Committee and conformed to the principles of the Declaration of Helsinki on human experimentation (WMADH, 2000).

### Testing procedures

The players were tested immediately after the season finished, during a two-week break. The assessment sessions were conducted over three separate days, between 9 and 11 a.m., with 48 hours between the sessions. To minimize variation in the climatic and other conditions, the shuttle run test was performed in a sport hall on a parquet floor. The Running-based Anaerobic Sprint Test (RAST) was performed on a track and field stadium. All players were familiarized with the testing procedures before the study commenced. All were encouraged to use as much effort as possible during all tests. A 10 min general warm-up (jogging), 7 min of active dynamic stretching and activities with progressive intensity (sprints and jumps) were performed before testing.

Players from each team were randomly split into two groups with an equal number of subjects. During the first testing day, body composition was assessed for each player. Afterwards, they performed the warm-up and a multistage shuttle run test, which was used to estimate maximal oxygen uptake (VO_2max_). On the second day, the players were tested using the countermovement jump to estimate their maximal anaerobic power. On the third day, the indices of anaerobic capacities were assessed using the RAST.

### Anthropometric data

To estimate the players’ anthropometric characteristics, the following variables were measured: body height (BH), body mass (BM), body fat content (BF%), and fat free mass (FFM%). Based on these measures, we calculated the body mass index (BMI) for each player (body mass (kg) / body height (m)^−2^). Body height was measured to the nearest 0.01 m with a portable stadiometer (Astra scale 27310, Gima, Italy). Body mass, body fat content and fat free mass were measured using a bioelectric body composition analyzer (Tanita TBF-300 increments 0.1%; Tanita, Tokyo, Japan).

### Vertical jump performance

The countermovement jump (CMJ) was assessed according to the protocol described by Bosco et al. (1983) and used to calculate the players’ anaerobic power. The subjects were asked to start from an upright position with straight legs and with hands on hips (to eliminate the contribution of an arm swing on jump height) and to perform a downward movement before the jump. They performed a natural flexion before takeoff. The participants were instructed to land in an upright position and to bend their knees on landing. Each player performed three maximal CMJ jumps, with 3 min of recovery between the jumps. The highest score was used for further analysis. The jumps were assessed using a portable device, called the OptoJump System (Microgate, Bolzano, Italy), which is an optical measurement system that consists of transmitting and receiving bars. Each of these contains photocells, which are positioned 2 mm from the ground. The photocells from the transmitting bar continuously communicate with those on the receiving bar. The system detects any interruptions in communication between the bars and calculates their duration. This makes it possible to measure the flight time and jump height during jump performance. The jump height is expressed in centimeters. The reliability of the CMJ in this study was very high (ICC = .91; α = 90; CV = 8.1%).

### Aerobic power

Maximal aerobic power (VO_2max_) was estimated using the 20 m shuttle run test according to Leger and Gadoury (1989). The test consisted of shuttle running at a pace preset by the shuttle run test protocol and played on a CD recorder. In the test, the participant ran 20 m long shuttles after a signal was sounded. At the start of the test, the subject had to run at a speed of 8 km/h to reach the opposite line before another signal was given. The running speed increased every minute by 0.5 km/h. When the subjects were unable to maintain the pace, the last covered shuttle was used to estimate VO_2max_. The test-retest reliability coefficients are 0.89 for children and 0.95 for adult men and women.

### Anaerobic capacity

Anaerobic capacity was assessed with the Running-based Anaerobic Sprint Test (RAST), which according to Zacharogiannis et al. (2004) could replace the Wingate test when estimating anaerobic power and capacity. Additionally, de Araujo et al. (2014) found that the RAST was a practical protocol for assessing the anaerobic index and was an excellent hyperlactatemia inductor. Each athlete performed a 12 min warm up (5 min of jogging and 7 min of active dynamic stretching), which was followed by 3 min recovery. The test consisted of 6 sets of 35 m intermittent sprints. Each sprint represented a maximal effort with 10 s allowed between each sprint for the turnaround. After completion of the test, the following variables were calculated: maximal power (MaxPOW), average power (AvePOW), minimal power (MinPOW), the fatigue index (FI) and relative maximal power (R-MaxPow). The variables were estimated by the following equations: Power = Weight (kg) × Distance (m^2^) ÷ Time (s^3^). Maximum power = the highest value of six sprints, minimum power = the lowest value of six sprints, average power = sum of all six values ÷ 6, fatigue index = (maximum power - minimum power) ÷ total time for the 6 sprints, and R-MaxPow = maximum power / weight. The test reliability (r = 0.90) was reported by Zagatto et al. (2009).

### Anaerobic power

Peak power and relative peak power output generated during the CMJ were calculated using two separate equations. The first was developed by Sayers et al. (1999) for estimating peak power output: PAPw (Watts) = (51.9 · height CMJ (cm)) + (48.9 · body mass (kg)) – 2007 and the second was derived from the first, and it represents relative peak power output standardized to the subject’s weight: R-PAPw (W/kg) = PAPw (watts) / mass (kg). Each player performed three maximal CMJs as described before with 3 min of recovery in between. The highest score was used for further analysis.

### Statistical Analysis

Descriptive statistics (mean, standard deviation, and range) were calculated for each variable. Data sets were checked for normality using the Kolmogorov-Smirnov test and by the visual observation of normality plots. Reliability and validity were assessed with an intraclass correlation coefficient (ICC) and a coefficient of variation (CV). The Levene’s test was used to assess homogeneity of variances. To determine the possible differences between the subjects who played at different positions, analysis of variance (one way ANOVA) with the Bonferroni post hoc test for multiple comparisons was used. The significance for all statistical tests was set at *p* ≤ 0.05. All statistical analyses were completed with the SPSS software statistical package (SPSS Inc., Chicago, IL; Version 14.0).

## Results

The results showed a normal distribution of the data and no violation of the homogeneity of variance. The results from one-way ANOVA indicated a significant difference in body mass and height among the groups. Post hoc tests using Bonferroni correction determined that centers were significantly (p < 0.01) heavier and higher than guards and forwards, while there was no significant difference between the groups in terms of body fat content, fat free mass and the body mass index (Table 1).

The aerobic and anaerobic power differences between the groups are displayed in Table 2. The Bonferroni post hoc test revealed that predicted VO2max was significantly higher (p < 0.01) in guards and forwards than in centers. The CMJ height and CMJ Relative Peak Power Output were higher (p < 0.01) in guards than in centers, while CMJ Peak Power Output was higher (p < 0.05) in centers. All RAST absolute values were higher (p < 0.01) in centers than in guards and forwards, while the relative values were higher (p < 0.01) in guards in comparison to forwards and centers. The RAST fatigue index was smaller (p < 0.01) in guards and forwards than in centers.

## Discussion

The main finding of this research was the existence of differences in aerobic and anaerobic indices between the players according to their positional role. Significant differences were found in eleven out of sixteen measured variables. These findings confirm the results of previous investigations conducted to compare players at different team positions in their aerobic and anaerobic variables (Abdelkrim et al., 2010; Gocentas et al., 2011; Ostojić et al., 2006; Sallet et al., 2005). Again, guards and forwards had higher aerobic power and higher values of relative anaerobic power and capacities, while centers displayed higher values of absolute anaerobic power.

VO2max was significantly higher in guards and forwards in comparison to centers, which can be explained by the specific requirements of their position on the court. Guards are excellent ball handlers who control the tempo of the game with high dribbling skills and fast transitions. This is supported by the research of Abdelkrim et al. (2007), who reported a greater number of actions performed and higher distance covered by guards compared to centers. Also guards and forwards spent significantly more time competing in high-intensity activities than centers. The importance of higher aerobic power in guards can be seen from the studies by Narazaki et al. (2009), who reported a positive correlation between VO2max and a number of high intensity activities, and Meckel et al., (2009), who also reported a positive relationship between a basketball-specific repeated sprint ability test and VO2max. Piiper and Spiller (1970) suggest that the aerobic energy system is important in the removal of lactate and in replenishing the creatine phosphate that supplies the body with energy for high-intensity activities. Moreover, the importance of aerobic power was emphasized by Štrumbelj et al. (2014), who reported VO2max to be the predictive attribute of young players’ current ability. A higher VO2max value enables guards to have a shorter recovery time between intensive bouts of defensive and offensive tasks (Hoffman et al., 1999; Tomlin and Wenger, 2001), while enabling them to efficiently repeat high intensity basketball-specific movements. Furthermore, higher VO2max in guards can be attributed to different training regimes of guards and forwards compared to centers. Guards spend more time performing high intensity full court drills, while centers are usually involved in half court drills with many contacts in the paint area. Our findings are in line with studies conducted by Köklü et al. (2011), Gocentas et al. (2011), Abdelkrim et al. (2010) and Ostojić et al. (2006). On the other hand, there is some discrepancy with the studies by Sallet et al. (2005) and Abdelkrim et al. (2007), who reported no significant differences in VO2max between different playing positions.

Considering the above finding, the guards had higher anaerobic capacities, according to the relative values obtained from the RAST test parameters, than the forwards and centers. They performed better in the relative maximal, average and minimal power output as well as in the ability to resist fatigue (FI - fatigue index). According to McInnes et al. (1995), the anaerobic energy systems are responsible for success in high intensity movements during a game. Knowing that guards have a higher frequency of sprints and shuffles than forwards and centers during a game (Abdelkrim et al., 2007; McInnes et al., 1995), it was expected that the guards would have a greater anaerobic potential.

In addition, the obtained superior relative anaerobic values by the guards are directly related to their team role and the size of their body compared to forwards and centers. Smaller body mass and dimensionality, along with a lower center of body mass, equip them with better ball handling, speed and agility with and without the ball. At the same time, centers’ greater body dimension limits their ability to effectively execute high intensity movements. These anthropometric characteristics, in combination with well-developed aerobic and anaerobic power and capacities, enable the guards to transition between high-intensity activities on the court with little or no recovery. Our findings can be supported by the results obtained by McInnes et al. (1995), Abdelkrim et al. (2007) and Erčulj et al. (2008), who reported that guards spent significantly more time competing at high-intensity (i.e., sprints, jumps, shuffles) than centers, and they had higher heart rate values and plasma lactate concentrations during a game.

On the contrary, the centers showed better results in absolute values of power output during the RAST and CMJ tests. This is understandable as centers in the present study were significantly heavier than guards and forwards, which supports previous studies (Abdelkrim et al., 2010; Jeličić et al., 2002; Ostojić et al., 2006; Sallet et al., 2005). Centers’ higher absolute power determines their role in the game i.e., a low-post position. Thus, they usually play near or inside the painted area, trying to obtain rebounds and score points close to the basket, while trying to block opponents’ shots. They use their body mass, body inertia, strength and power to efficiently complete their specific positional tasks during the games, all of which involve substantial contact with the opponents. Higher absolute power enables them to efficiently grab rebounds, set screens and box-out opponents.

In addition, there were significant differences in the CMJ heights between the groups; the guards achieved better jump height compared to the centers. These results are in line with recent studies (Abdelkrim et al., 2010; Hoare, 2000; Köklü et al., 2011; Ostojić et al., 2006) reporting similar results between different positional roles, but the fact is that players from the Bosnian league achieved lower values in the CMJ height compared to some previous studies (Abdelkrim et al., 2010; Hoffman et al., 1996; McInnes et al., 1995; Ostojić et al., 2006). On the other hand, there were no significant differences in the CMJ performance between players from this study and a study performed by Köklü et al. (2011). It can be observed that all three groups of players had well-developed aerobic power, which was even better than values reported by some recent studies (Abdelkrim et al., 2010; Köklü et al., 2011; Ostojić et al., 2006). This is most likely because subjects from the current study had smaller and lighter bodies compared to the international elite players examined in recent studies.

However, there were some discrepancies when comparing the morphological results of the present study to previous data. Although the values of body composition, the body mass index, body fat percentage, and fat free mass were higher in centers than in other groups, there were no significant differences between them. All in all, these small differences between the groups in body composition variables can be attributed to the well-designed strength and conditioning programs of all players, which decrease the chances of body fat being stored differently in players according to their team role. Furthermore, all groups of players participating in the present study were shorter and lighter than those measured in previous studies (Abdelkrim et al., 2010; Köklü et al., 2011; Ostojić et al., 2006; Sallet et al., 2005). A possible explanation for this difference may be the players’ age as our subjects were younger when compared to those from other studies. It is likely that they did not reach maturity and would continue to mature after the age of twenty (Tanner, 1962). Additionally, the present study showed similar results for body fat content and fat free mass for guards and forwards as well as some higher values for the centers compared to more recent studies (Abdelkrim et al., 2010; Köklü et al., 2011; Ostojić et al., 2006; Sallet et al., 2005).

Limitation of the study is that the subjects were tested immediately after the season finished which might have influenced the results due to fatigue and loss of motivation. Other limitations included the lack of heart rate and blood lactate concentration evaluations across testing procedures which could help in explaining the underlying mechanisms of the physiological load imposed during the assessments.

## Conclusions

The findings of the present study suggest that aerobic and anaerobic power and capacities can be good discriminative variables between players with different positional roles. Guards and forwards had better aerobic and relative values of anaerobic power, which provided them with a shorter recovery time and ability to efficiently repeat high intensity basketball-specific activities. Centers had higher values of absolute anaerobic power and capacities, equipping them with the ability to play powerfully with substantial contact.

During training sessions, coaches can use this information to create more individualized strength and conditioning programs for different positional roles. This will enable them to maximize the players’ physiological potential, which is an integral part of playing basketball. These data may be more useful, especially to Bosnian basketball coaches and players as only few studies have investigated the physiological profile of Bosnian basketball players. The results suggest the importance of introducing more extensive and intensive plyometric training programs to improve explosive power as well as the selection of players with higher values of anaerobic power potential.

## Figures and Tables

**Table 1:** Mean and SD (range) of the age and morphological characteristics for guards, forwards and centers

VARIABLES	GUARDS (n = 22)	FORWARDS (n = 19)	CENTERS (n = 14)
Age (years)	19.36 ± 3.54 (16–28)	18.21 ± 2.65 (17–25)	19.86 ± 2.98 (16–27)
Body height (cm)	182.88 ± 6.10 (171.0–191.6)*†	190.02 ± 6.58 (176.0–201)†	197.75 ± 4.40 (191.8–206.2)
Body mass (kg)	77.38 ± 11.36 (58.8–96.6)†	81.48 ± 9.33 (68.0–100.9)†	95.55 ± 9.61 (77.7–112)
Body fat (%)	12.41 ± 4.19 (6.1–19.5)	12.28 ± 3.05 (8.1–17.8)	15.04 ± 4.64 (5.5–24.2)
Fat free mass (%)	87.58 ± 4.18 (80.5–93.88)	87.70 ± 3.05 (82.14–91.88)	84.94 ± 4.64 (75.8–94.47)
BMI (kg ·m^−2^)	23.06 ± 2.61 (18.8–27.0)	22.57 ± 2.49 (18.1–28.5)	24.60 ± 2.76 (19.4–30.4)

* values significantly different from those obtained by forwards; p < 0.05.

† values significantly different from those obtained by centers; p < 0.05.

**Table 2:** Mean and SD (range) of the aerobic and anaerobic indices for guards, forwards and centers

VARIABLES	GUARDS (n = 22)	FORWARDS (n = 19)	CENTERS (n = 14)
VO2max (ml · kg^−1^ · min^−1^)	64.36 ± 7.05 (40.84–76.41)†	62.38 ± 6.08 (50.72–74.43)†	57.91 ± 7.23 (46.77–69.0)
Number of shuttles completed (n)	105.64 ± 14.28 (68 – 130)†	101.63 ± 12.31 (78 – 126)†	92.57 ± 14.65 (70 – 115)
CMJ height (cm)	40.40 ± 5.04 (33.3–53.6)†	37.62 ± 6.80 (29.4–55.4)	36.04 ± 3.80 (29.3–44.3)
RAST Maximal Power (Watts)	772.96 ± 129.38 (579.7–998.8)	762.20 ± 129.50 (572.5–1044.2)	858 ± 108.92 (595.8–986.5)
RAST Minimum Power (Watts)	513.14 ± 109.16 (365.7–750.1)	471.15 ± 73.65 (377.3–589.3)	531.9 ± 83.47 (371.1–659.8)
RAST Average Power (Watts)	634.87 ± 109.64 (451.7–839.1)†	607.47 ± 89.62 (479.1–808.2)†	712.65 ± 69.45 (579.5–795.8)
RAST Fatigue Index (Watts/s)	8.08 ± 2.49 (4.68–13.33)†	8.83 ± 2.67 (4.95–13.45) †	10.48 ± 2.24 (6.70–13.99)
RAST Relative Maximal Power (Watts/kg)	14.90 ± 1.08 (12.78–16.95)†	13.94 ± 1.45 (11.40–17.04)	13.44 ± 1.46 (8.96–14.98)
RAST Relative Minimal Power (Watts/kg)	9.89 ± 1.42 (7.49–13.0)*†	8.62 ± .89 (7.14–10.29)	8.31 ± 1.08 (7.13–10.52)
RAST Relative Average Power (Watts/kg)	12.24 ± 1.13 (10.62–14.48)*†	11.12 ± .93 (9.43–12.94)	11.15 ± .70 (9.99–12.41)
CMJ Peak Power Output (Watts)	3874.42 ± 639.3 (2740.50–5005.53)†	3930.4 ± 604.1 (2951.25–4945.92)†	4536.4 ± 458.4 (3775–5379)
CMJ Relative Peak Power Output (Watts/kg)	50.02 ± 3.46 (44.93–60.02)†	48.16 ± 4.33 (43.02–59.73)	47.51 ± 2.06 (43.94–52.14)

* values significantly different from those obtained by forwards; p < 0.05.

† values significantly different from those obtained by centers; p < 0.05.
